# How Does Positive Work-Related Stress Affect the Degree of Innovation Development?

**DOI:** 10.3390/ijerph17020520

**Published:** 2020-01-14

**Authors:** Gema Albort-Morant, Antonio Ariza-Montes, Antonio Leal-Rodríguez, Gabriele Giorgi

**Affiliations:** 1 Departamento de Economía Financiera y Dirección de Operaciones, Universidad de Sevilla, 41018 Sevilla, Spain; galbort@us.es; 2 Departamento de Gestión Empresarial, Universidad Loyola Andalucía, 14004 Córdoba, Spain; 3 Facultad de Administración y Negocios, Universidad Autónoma de Chile, Santiago 425, Chile; 4 Departamento de Administración de Empresas y Marketing, Universidad de Sevilla, 41018 Sevilla, Spain; aleal6@us.es; 5 Department of Human Sciences, Università Europea di Roma, 00163 Roma, Italy; prof.gabriele.giorgi@gmail.com

**Keywords:** work-related stress, innovation, partial least squares

## Abstract

Many studies sustain that work-related stress exerts pervasive consequences on the employees’ levels of performance, productivity, and wellbeing. However, it remains unclear whether certain levels of stress might lead to positive outcomes regarding employees’ innovativeness. Hence, this paper examines how the five dimensions of work-related stress impact on the employees’ levels of innovation performance. To this aim, this study focused on a sample of 1487 employees from six Italian companies. To test the research hypotheses under assessment, we relied on the use of the partial least squares (PLS) technique. Our results reveal that, in summary, the stressors job autonomy, job demands, and role ambiguity exert a positive and significant impact on the employees’ levels of innovativeness. However, this study failed to find evidence that the supervisors’ support–innovation and colleagues’ support–innovation links are not statistically significant.

## 1. Introduction

Work-related stress, also known as job stress, is frequently defined as a feeling of work-related hardness, frustration, distress, or tension [1,2]. It is the result of numerous negative effects in the work environment (e.g., deviation from normal physical and psychological functioning as absenteeism, unhappiness, tension, anxiety, turnover intent, or burnout among others). However, several authors suggest that work-related stress also causes positive effects on organizational outcomes such as performance, job satisfaction, organizational commitment, or degree of innovation [3,4,5]. In this study, we focused on the relationship between work-related stress and innovation. According to several empirical studies, hindrance stressors, such as role conflict, job ambiguity, and role overload, could serve as a motivational source to promote the implementation of individual innovative behavior [6]. Therefore, this study posits that those workers who feel stressed can generate new ideas due to the necessity to modify oneself or the work environment.

This paper is in line with the study developed by [7], who presented detailed information about the development, reliability, and validity of the Stress Questionnaire (SQ), which is based on five main psychosocial risks that might lead to stress-related negative outcomes. In this study, we focused on the five subscales of work-related stress: (a) job demands; (b) job control; (c) role ambiguity; (d) supervisors’ support; and (e) colleagues’ support proposed by these authors. These five factors or subscales of work-related stress, commonly labeled as “job stressors”, have been studied independently by other authors. However, this study addresses them jointly to assess how they influence employees’ levels of innovativeness.

To date, relatively few studies have addressed the linkages between job stressors and innovative behavior [8]. Several studies have analyzed the relationship between a certain job stressor and innovativeness in isolation. For instance, a study developed by [9] examined the relationship between role conflict and innovation, while others examined the link between colleagues support and innovation [10], supervisor support and innovation [11,12,13,14], job demands and innovation [15,16,17], or job control or job autonomy and innovation [18,19,20,21]. However, to our knowledge, no study has intended to assess the joint effect of these five stressors on the employees’ innovative behavior or jointly linked each of these five dimensions that shape work-related stress with innovation.

Therefore, the purpose of this study was to offer a decisive contribution to the field by gathering work-related stress and innovation literature and exploring how certain levels of work-related stress may entail interesting implications for employees’ innovativeness. To test the research model and hypotheses, this study relied on the use of partial least squares (PLS) path-modeling, a variance-based structural equation modeling technique that was applied to a sample of 1487 Italian employees. This study was focused in the context of Italian companies that are characterized by product development cycles that are constantly updating.

The following paper is organized as follows. Section 2 brings a thorough review of the literature on work stress and innovation to identify and measure each of the constructs that constitute the research model under assessment. This section also presents a research model that posits a direct link between work stress dimensions (role ambiguity, colleagues’ support, supervisors’ support, job demands, and job autonomy) and innovation. Section 3 describes the sector overview and the methods applied in this study. Section 4 presents the empirical results derived from PLS analyses. Finally, Section 5 concludes with a discussion of the empirical results, and their implications for management practitioners.

## 2. Conceptual Framework

### 2.1. Work-Related Stress

While a variety of definitions of the term work-related stress have been suggested, this paper uses the definition suggested by Giorgi et al., [7] which is based on five subscales of this term: (a) job demands; (b) job control; (c) role ambiguity; (d) supervisors’ support; and (e) colleagues’ support. Below, the definitions for the five above-mentioned job stressors are provided.

### 2.2. Colleagues’ Support

Colleagues’ support, also known as coworker support, is based on the theory of social exchange. It deliberates that employees tend to establish long-term social exchanges among partners, such as their coworkers, to encourage the collaboration ties and emotional support among them [22].

The term colleagues’ support is defined as “the extent to which employees believe their coworkers are willing to provide them with work-related assistance to aid in the execution of their service-based duties” [23]. Colleagues’ support enables the share of knowledge and experience between colleagues who have recently joined their job or who need help with difficult, challenging, or new tasks. They have a similar role, experience, position, hierarchical level, and reach a common goal [10]. Employees know what their coworkers may feel because they have passed through the same situation. Hence, employees believe they can look for help from peers [24], and, at the same time, they feel obligated toward the team [25]. The support in reciprocal manner will turn into their philosophy. They will also help to develop unified teams where everyone knows each other.

Furthermore, this concept also includes socioemotional support such as care, empathy, and affection [26] that could favor the increase of employee work satisfaction and subjective well-being. In this way, colleagues’ support will encourage employees to cooperate to develop new and creative ideas—something that would not happen if there exist competitive attitudes, tension, and less verbal communication among colleagues at the workplace [27,28].

### 2.3. Job Autonomy

Autonomy can be broadly defined as “the central work characteristic in shaping worker attitudes, motivation and behavior” [29]. In this line, job autonomy (control over work) refers to the own decisions about their scheduling, procedures, and work-related tasks [30]. This term implies some dimensions, such as when, where, how, and at what incomes a duty is developed [31]. Therefore, employees have substantial independence and freedom to leverage their working hours besides the individual decisions that they take to carry out their main functions. The free work condition and the absence of procedures and rules may influence the degree of employee’s proactivity actions [20]. This will allow workers to develop new and innovative processes and products. However, this only happens when hierarchical links with the organization are clear and transparent and employees’ opinions and suggestions are not limited [32].

Several studies have considered the positive outcomes of job autonomy as greater job satisfaction, proactivity, and increased well-being, supporting personal motivation, innovation and increase self-esteem [18,32,33,34,35,36,37]. Similarly, high levels of job autonomy also relate to low levels of stress outcomes such as burnout, anxiety, and irritability, among other symptoms [38]. They present a definition that is based upon engagement in behaviors, regardless of the ultimate outcomes.

### 2.4. Job Demands

The term job demands have come to be used to refer to a high workload required to employees (individually or in teams). LePine et al., [39] argued that job demands need employees to work hard and fast as well as manage a high quality of work. The authors of [40] defined the term as “those physical, psychological, social, or organizational aspects of the job that require sustained physical and/or psychological (cognitive and emotional) effort or skills and are therefore associated with certain physiological and/or psychological costs” (p. 312).

Thus, they showed that this term is a specific psychological stressor. When people work in a high physical demand, high time pressure, low decision latitude, emotional demands, role ambiguity, unfavorable physical environment, tension with coworkers, shift work, or less adequate procedures [40,41], people may develop psychological and chronic problems, e.g. sleeping problems, burnout, and impaired health [42]. In this line, employees and managers should work together in the development of a problem-focused strategy. To do this, they should cultivate new and innovative working methods to improve and increase the effective response to the demands and high-quality in a determinant context.

### 2.5. Role Ambiguity

Role ambiguity can be defined as the amount of uncertainty perceived by a person responsible for a specific activity regarding what exactly should he or she do or accomplish [43]. This conceptualization is in line with the view of Mañas et al., [44], who relied on Kahn et al., [45] to define role ambiguity as “the lack of clarity in understanding the actions to be taken to achieve proposed individual goals”. It is certain that the existence of ambiguity regarding the goals, objectives, and procedures may affect how the employees understand their function and what they are supposed to do at the workplace or how their performance will be evaluated. This might seriously contribute to a decrease in employees’ performance and goals attainment levels [46].

### 2.6. Supervisors’ Support

Drawing upon social exchange theory, supervisors’ support and management support have been defined by a growing body of authors over the years. Among them, the authors of [47] described supervisor’s support as “the extent to which employees perceived that their supervisors afforded them flexibility and freedom, encouraged their suggestions and opinions, and provided opportunities for training” (p. 33). Supervisor is understood as more experienced workers or immediate supervisors who serve teaching and evaluative daily work functions [10]. However, managerial support does not always have the same attitude in relation to their employees. In this line arises the abusive supervision, which could have a negative impact and serious consequences in organization. This term refers to the subordinates’ perceptions about sustained display of hostile verbal and nonverbal behaviors of their subordinates [48]. This could lead to an unethical leadership due to supervisors violates normative standards of behavior with their employees (e.g., when humiliating and ridiculing employees) [14]. Another example would be that abusive supervision will affect work teams given that the actions of leader may have a systematic effect on all members. They feel conditioned by authority and status of supervisor [14,49].

Therefore, it would be advisable that managers try to build a supportive environment in which employees receive support from supervisors. Employees understand their responsibilities and duties through frequent communication, training, or team building sessions [25]. Encouraging the development of innovations, creativity, responsiveness to change, and sharing of information and resources [47] will increase job satisfaction, enhance professional psychological health, facilitate employee’s performance, and reduce turnover and absenteeism.

## 3. Innovation

Following prior research, we define innovation as “a means of changing an organization, either as a response to changes in the external environment or as a pre-emptive action to influence the environment. Hence, innovation is here broadly defined to encompass a range of types, including new products or services, new process technology, new organization structure or administrative systems, or new plans or programs pertaining to organization members” [50]. In other words, innovation is the generation, approval, and application of new ideas, products, services, or processes.

Therefore, considering the widely accepted view that innovation is defined as the translation of ideas into something lucrative, encouragement to supply ideas needs to be substantial to channel the creative skill of the workers to transform ideas into innovations [51]. In particular, employees’ innovative behavior in the workplace will be generated if they are happy with the organizational development. Therefore, managers should motivate their employees to be more proactive, creative, and innovative without damaging their psychological health.

### Work-Related Stress and Innovation

In recent years, one of the main concerns of organizations is the increase and impact of work-related stress in the degree of individual productivity and performance of its employees. A small variation of work-related stress and innovation performance (both at the same time) will translate into huge income or lost for organizations [8]. However, although there are studies that assess the high impact that work stress exerts on individuals’ productivity or performance decrease, there is a scarcity of research aimed at assessing the influence of work-related stress on the employees’ innovation performance. Given that nowadays more than ever innovation is considered a key factor while defining an organization’s survival within the current hypercompetitive environment, it is worth investigating which factors might contribute to enable or hinder employees’ innovativeness.

Following Lazarus [52], work stress emanates from an individual’s perception of external demands as more difficult or beyond its own perceived competencies. Hence, in line with the work stress and strain framework, work stressors are the factors or stimuli underlying the stress process, which often lead individuals to suffer from strain, anxiety, turnover, absenteeism, or burnout, among other consequences [53,54]. However, recent studies revealed that not all the stressors lead to the same consequences and that they frequently lead to distinct behaviors among individuals. For instance, the authors of [55] argued that there are two kinds of stressors challenge stressors and hindrance stressors) depending on whether such stressors were interpreted by the individual as opportunities for learning and growing or as obstacles for personal growth and achievement. In this vein, several studies have found that both types of stressors impact differently on individuals’ behaviors at the workplace [39,56].

Therefore, this paper argues that certain levels of work-related stress might contribute to enhance individuals’ levels of innovativeness at the workplace. Hence, the above-mentioned work stressors, at certain levels, might enable the innovation process. For instance, under circumstances or scenarios characterized by high job demands (workload), employees may need to act to develop new and innovative ways of working [17] in order to comply with such demands. This is in line with the person–environment fit theory [57], which states that innovative behavior might assist employees aimed at enhancing their fitting with highly demanding scenarios at the workplace.

In the same way, job autonomy may exert a significant effect on workplace innovation since it might allow employees to develop their tasks independently, and hence, they can think, reflect, and come up with new or innovative ways of complying with their duties [18]. This is a job characteristic that provides different combinations of work methods [58]. In this way, employers feel a responsibility to get out of the routine work and try for a better solution [59]. Therefore, employers with higher job involve innovative behavior [60].

Similarly, role ambiguity about the job’s content-related issues might play a beneficial role in innovation. For employees to achieve their work objectives, they must seek effective and creative solutions (innovations), while feedback from colleagues reduces the uncertainty and problems resulting from the uncertainty and unclear roles [61].

Besides, the existence of close relationships and accurate feedback among subordinates or colleagues might be also positive, since it can contribute to the generation of new, fresh, and useful ideas [62]. According to the authors of [60,63], healthy relations can accommodate the preferences and demands of the clients by customizing their products and services in a creative and innovative fashion. In addition, managers must promote a cooperative climate whose group relations (coworkers and supervisors) are based on trust, openness, not jealousy, good communication, and positive lead.

**Figure 1:**
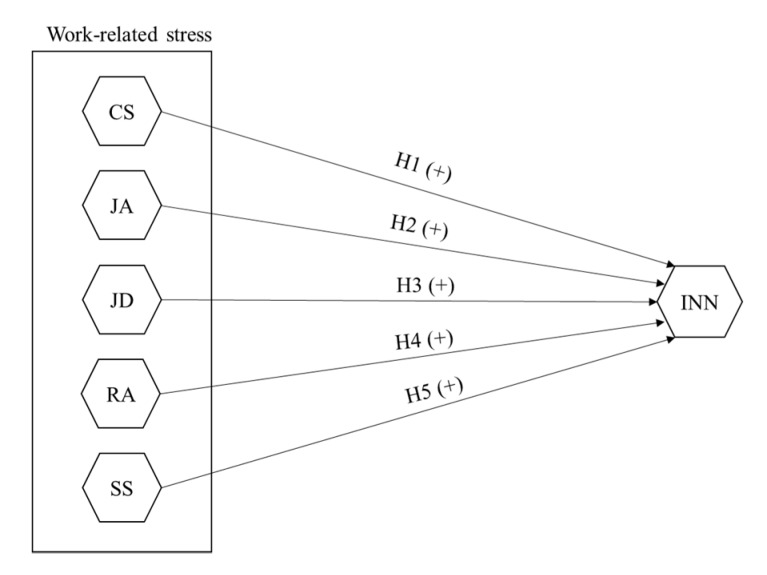
Research model and hypotheses. Notes: CS, Colleagues’ Support; JA, Job Autonomy; JD, Job Demands; RA, Role Ambiguity; SS, Supervisors’ support; INN, Innovation. Source: Own elaboration.

According to mentioned literature, we posit the following research hypotheses (please, see Figure 1):

**Hypotheses 1** **(H1).** *There is a positive relationship between employees’ perception of colleagues’ support and innovation*.

**Hypotheses 2** **(H2).** *There is a positive relationship between employees’ perception of job autonomy and innovation*.

**Hypotheses 3** **(H3).** *There is a positive relationship between employees’ perception of job demands and innovation*.

**Hypotheses 4** **(H4).** *There is a positive relationship between employees’ perception of role ambiguity and innovation*.

**Hypotheses 5** **(H5).** *There is a positive relationship between employees’ perception of supervisor support and innovation*.

## 4. Methodology

### 4.1. Data Collection and Sample

The data for this research were collected over the period 2017–2018 by a team of researchers who examined demographic variables, work related stress, innovation, and welfare, among other constructs. The final sample obtained consists of 1487 employees from six Italian companies on a multi-sectorial basis (fashion design industry, construction industry and ice cream industry). The participants had one hour to complete the questionnaire with paper and pencil through a guided administration. Only subjects from the Knauf company completed the online questionnaire using an online survey platform called SurveyMonkey (SurveyMonkey Inc., San Mateo, CA, USA). The subjects were informed about the objectives of the research and data protection. The anonymity of the subjects has been ensured through the use of barcodes.

### 4.2. Measures

This work used 25 items from the Stress Questionnaire, a previously used and validated scale developed by Giorgi et al., [7], to measure work-related stress that include five stress-related factors or dimensions: (a) role ambiguity (e.g., “I understand how my work is functional to the general goals of my organization”); (b) colleagues’ support or collaboration and support among employees (e.g., “my colleagues are willing to listen to problems concerning work”); (c) supervisors’ support or understanding from their supervisors/leaders (e.g., “I am encouraged by my manager/supervisor”); (d) job demands (e.g., “I have unreasonable deadlines”); and (e) job control or autonomy (e.g., “I have some leeway in deciding what to do at work”). This study used a five-point Likert scale from absolutely agree to absolutely disagree to measure the questionnaire items. Similar to many questionnaires that employ a Likert-type scale for answering questions, with the purpose of minimizing response style bias, this survey instrument contains some items which are reverse scored. Reverse scoring implies that the numerical scale runs in the opposite direction. Thus, for instance, the “strongly disagree” answer would be assigned the maximum value of the scale, while “strongly agree” answer would be assigned the minimum value of the scale. The factorial structure of this questionnaire has been supported by the same authors in other previous and later studies [64,65].

The innovation variable consists of nine items instead of the traditional single dimensional measures. To this aim, this study adapted the nine items from the scale proposed by Janssen [15], which measures the frequency with which employees claim to be involved in the generation, promotion, and implementation of new ideas in the workplace [15]. The survey items shaping both measurement scales have been fully included within the Appendix A.

### 4.3. Data Analysis

To empirically test the research hypothesis proposed, this study relied on Partial Least Squares (PLS) path-modeling, a variance-based structural equation modeling (VBSEM) technique [66]. This technique was chosen mainly because all the constructs comprised at the conceptual model are modeled as composites. Many theoretical and empirical studies recommend the use of PLS in cases of composite measurement model [67]. Furthermore, this study was essentially focused on the prediction of the dependent construct—Innovation [68]. The exogenous constructs—RC, CS, SS, JD, and JA—were modeled as composite constructs estimated in Mode B (regression weights), while Mode A (correlation weights) was selected to measure the endogenous construct—INN. Besides, SmartPLS 3.2.7 software was used to conduct the data analysis [69].

## 5. Results

### 5.1. Evaluation of the Measurement Model

The assessment of the PLS measurement model displays satisfactory results. First, regarding the Innovation construct, it was modeled as a composite construct and estimated in Mode A. This involved the assessment of the measurement model by the following sequential steps: (i) individual item reliability; (ii) construct reliability; (iii) convergent validity; and (iv) discriminant validity. All indicators measuring the Innovation construct comply with the requisite of individual item reliability, given that all the outer loadings surpass the 0.707 threshold (Table 1). Similarly, this construct satisfies the requirements of construct reliability (Cronbach’s Alpha and Composite Reliability are greater than 0.7 (Table 1)) and convergent validity (Average Variance Extracted (AVE) is over the 0.5 critical level (Table 1)). Lastly, Table 1 reveals that discriminant validity is achieved, according to the heterotrait–monotrait ratio (HTMT) criterion that specifies that values must be under the threshold of 0.85 [70].

Second, the RA, CS, SS, JD, and JA constructs were been modeled as composite constructs estimated in Mode B. Thus, they were assessed in terms of: (i) potential multicollinearity between items; and (ii) weight assessment [66]. In line with what the authors of [71] recommended, variance inflation factor (VIF) values over the threshold of 3.3 imply the existence of high multicollinearity between items. However, the authors of [69] claimed that multicollinearity should be a serious concern only when VIF values surpass the critical level of 5. In our case (Table 1), the maximum VIF value for indicators ascends to 2.492, well below the critical levels proposed by both studies [69,71]. Therefore, multicollinearity does not seem to be a concern in this study. Next, it was compulsory to assess the magnitude and significance of the weights (Table 1). Weights provide information regarding how each indicator contributes to form the respective composite [72], allowing to rank the indicators based on such contribution.

### 5.2. Evaluation of the Structural Model

As recommended by Hair et al., [68], this study employed a bootstrapping (5000 resamples) technique to generate the standard errors, t-statistics, *p*-values, and 95% bias corrected confidence intervals (BCCI) that allow evaluating the direction and statistical significance of the relationships hypothesized within the research model. Table 2 includes the key parameters obtained for the structural model under assessment in this study. The coefficient of determination (R^2^) was used as the principal criterion for measuring explained variance—the extent to which exogenous constructs explain the endogenous one. The results in Table 2 reveal that the structural model reaches satisfactory predictive relevance for the endogenous construct, since R^2^ = 0.275 (Table 2). However, not all the direct relationships hypothesized appear to be statistically significant. While this study found empirical support for Hypotheses H2–H4, Hypotheses H1 and H5 are not supported.

### 5.3. Evaluation of the Predictive Ability of the Model

This study also verified if the proposed research model has predictive ability. With this regard, the authors of [73] stated that a model’s predictive performance is its ability to produce precise predictions of new observations, being them either temporal or cross-sectional in nature. In this vein, the authors of [74] claimed that explanation and prediction encompass two distinctive goals that might be combined in a research study. This view is supported by Dolce et al., [75], who posited that “The predictions of path models should be sensitive to the theory. In particular, the theoretical model represented by the structural equations and prediction should not be separated”.

Therefore, this study analyzed the conceptual model’s predictive ability (out-of-sample prediction) by means of cross-validation with holdout samples [76] focusing on the key endogenous construct (INN). Precisely, this study relied on the use of the PLS predict algorithm [77] offered in the SmartPLS 3.2.7 version [69]. To assess if the proposed model attains predictive ability, it was mandatory to verify whether the *Q*^2^ values are greater than 0, which would infer that the prediction errors of PLS outcomes are smaller than the prediction errors of merely using the mean values. The model proposed in this study complies with this criterion at both the construct (LV Prediction Summary) and indicators (MV Prediction Summary) levels (Table 3).

## 6. Conclusions

Building upon the prior-related scientific literature [39,55,56], this study developed a research model that links the main five work-related stressors (i.e., colleagues’ support, job autonomy, job demands, role ambiguity, and supervisors’ support) with the employees’ innovation performance. Thus, this study argued that certain levels of work-related stress might contribute to enhance or drive employees’ levels of innovativeness at the workplace. Therefore, work-related stress need not always be negative. Meanwhile, work-related stressors can favor or impede an innovative job, which is necessary to stay in the labor competition.

This paper contributes to the theory and practice by explaining the role of work-related stressors on the employees’ innovativeness at their workplace. After testing the research hypotheses by means of the application of the PLS technique and using a sample made up of 1487 employees belonging to Italian firms, our results reveal that the stressors job autonomy, job demands, and role ambiguity exert a positive and significant impact on the employees’ levels of innovativeness. Thus, this study offers empirical evidence to support the proposed research Hypotheses H2–H4. However, this study failed to find evidence to support Hypotheses H1 and H5, given that the supervisors’ support–innovation and colleagues’ support–innovation links are not statistically significant. This circumstance may perhaps be motivated by the fact that social support does not directly impact on innovation (i.e., direct link), but rather it exerts a moderating effect on the job demands–innovation relationship, in line with what the job demands–control–support model posits, a theory that continues to be highly influential in occupational stress and health literature [78].

Considering these findings, contrary to many studies that posit that work-related stress leads to negative outcomes, we may conclude that certain levels of work-related stress exert a positive influence on employees by means of stimulating them to seek for innovativeness. Positive or good stress (also known as eustress) stimulates us to face our daily problems and challenges, constituting the catalyst that allows workers to be creative, to take initiatives, and to respond efficiently to the demands that require it. It is in this sense that the authors of [79] showed that a certain type of stress might constitute a good mechanism to improve performance and innovation. Thus, this positive stress is a good way to face challenges and achieve better results for employees’ working lives. It should not be a continuous stress that could lead to the development of burned-out worker syndrome, but rather activation highlights during your working day. The physical activation that comes with short-term and punctual stress facilitates concentration, creativity, and innovation because it makes you more alert.

Besides, as shown in Table 3, the research model under assessment in this study entails predictive ability at both the construct (LV Prediction Summary) and the indicators (MV Prediction Summary) levels, which means that the stressors accurately predict new observations of the endogenous construct, being them either temporal or cross-sectional in nature.

### 6.1. Implications for Managerial Practice

This study has significant practical implications for human resources professionals, in particular, and managers in general. First, the empirical results suggest that, to improve the employees’ levels of innovativeness, organizations should provide them with a challenging context at the workplace. In particular, those responsible for human resources management should be aware (and in many cases convince managers of other functional areas and, even, senior or top managers) that caring for the work environment is a critical factor to stimulate the innovation capacity of workers. Providing employees with the correct levels of job autonomy, job demands, and role ambiguity to encourage their divergent thinking and creativity should be the way to go if firms aim to boost their employees’ levels of innovativeness. It should not be disregarded that innovation implies risk, and risk increases the probability of error. Zero tolerance for errors limits the creativity and innovative potential of workers. Thus, managers must understand that job autonomy must be based on a climate of trust where mistakes are not regarded as a synonym of failure, but rather as an opportunity for learning. Job autonomy will only be effective in this scenario, and only then will companies have more disruptive and innovative workers.

### 6.2. Limitations and Future Lines of Research

This work has limitations that offer opportunities for further research. Firstly, the sample is made up of companies belonging to different sectors and within a particular geographical context (Italy). Hence, researchers should not generalize the results. Further research could compare different sectors or expand the sample with employees from other European companies. Secondly, the research model only includes direct hypotheses. In future research, the indirect effect of variables related to the working atmosphere, company’s facilities, timetables, relations with other companies, etc. should be measured. The third limitation concerns individual perceptions, and we use only a single method to elicit these perceptions. Thus, a case study could perhaps be useful, as it would provide us with qualitative data and insights that could be helpful to improve manager’s implications. In addition, a 360-degree analysis could be made to get to know the different points of view of the employees. Finally, it might be interesting to investigate in depth the degree of positivity or negativity of each work stressor. In this sense, future studies should link the Inverted-U theory of stress, on the one hand, with the factors that constitute barriers to personal enhancement (hindrance stressors), and, on the other hand, with those factors that suppose an opportunity for learning and growth (challenge stressors).

## Figures and Tables

**Table 1:** Measurement model assessment.

Construct/Indicators	Outer Loadings	Outer Weights	VIF	Cronbach’s Alpha	Composite Reliability	AVE
Innovation (INN)				0.960	0.965	0.754
In1	0.851	0.095				
In2	0.792	0.084				
In3	0.873	0.097				
In4	0.848	0.128				
In5	0.890	0.144				
In6	0.927	0.142				
In7	0.855	0.157				
In8	0.871	0.138				
In9	0.900	0.162				
Colleagues’ support (CS)				N.A.	N.A.	N.A.
cs1	0.739	0.263	1.521			
cs2	0.726	0.198	1.707			
cs3	0.809	0.338	1.827			
cs4	0.850	0.361	2.046			
cs5	0.597	0.136	1.438			
Job autonomy (JA)				N.A.	N.A.	N.A.
ja1	0.680	0.502	1.369			
ja2	0.409	0.082	1.278			
ja3	0.543	0.244	1.343			
ja4	0.573	0.323	1.300			
ja5	0.751	0.410	1.283			
Job demands (JD)				N.A.	N.A.	N.A.
jd1	0.759	0.275	1.696			
jd2	0.552	0.113	1.314			
jd3	0.656	0.045	1.778			
jd4	0.677	0.136	1.601			
jd5	0.895	0.492	1.956			
jd6	0.829	0.202	2.492			
Role ambiguity (RA)				N.A.	N.A.	N.A.
ra1	0.397	0.045	1.218			
ra2	0.856	0.549	1.941			
ra3	0.431	−0.103	1.401			
ra4	0.895	0.622	1.458			
Supervisors’ support (SS)				N.A.	N.A.	N.A.
ss1	0.630	0.345	1.167			
ss2	0.743	0.303	1.466			
ss3	0.676	0.197	1.425			
ss4	0.848	0.501	1.591			
**Discriminant validity: Heterotrait–Monotrait Ratio (HTMT)**						
Construct	CS	CA	JD	RA	SS	
Innovation	0.487	0.544	0.517	0.480	0.445	

Note: VIF, Variance Inflation Factor; AVE, Average Variance Extracted; N.A., Not Applicable.

**Table 2:** Structural model results.

Relationship	Coefficient of Determination	Path Coefficient	T Statistics	*p*-Value	95% BCCI	Support
H1: Colleagues’ support → Innovation	R^2^_Innovation_ = 0.275	−0.015	0.186	0.852	[−0.198; 0.141]	No
H2: Job autonomy → Innovation		0.126 *	1.650	0.104	[0.004; 0.293]	Yes
H3: Job demands → Innovation		0.358 **	2.398	0.017	[0.049; 0.616]	Yes
H4: Role ambiguity → Innovation		0.157 *	1.728	0.085	[0.011; 0.398]	Yes
H5: Supervisors’ support → Innovation		−0.067	1.156	0.248	[−0.228; 0.018]	No

Notes: t values in parentheses. Bootstrapping 95% bias corrected confidence intervals (based on n = 5000 subsamples). ** *p* b 0.01; * *p* b 0.05.

**Table 3:** Predictive performance summary.

**LV Prediction Summary**			
	RMSE	MAE	*Q* ^2^
Innovation	0.686	0.110	**0.278**
**MV Prediction Summary**			
	*Q*^2^ PLS	*Q*^2^ LM	*Q*^2^ PLS-LM
In1	0.061	0.173	−0.112
In8	0.186	0.090	0.096
In7	0.240	0.206	0.035
In9	0.255	0.224	0.031
In6	0.194	0.144	0.050
In5	0.200	0.140	0.060
In3	0.054	0.085	−0.031
In4	0.160	0.126	0.034
In2	0.033	0.102	−0.069

Notes: RMSE, Root mean squared error; MAE, Mean absolute error; PLS, Partial least squares path model; LM, Linear regression model.

## Appendix A

Questionnaire items:

Social support

▪ ss1. I can count on the help of my supervisor/manager when a work problem arises.
▪ ss2. I am encouraged by my manager/superior.
▪ ss3. My supervisor is neither very competent nor very sure of himself.
▪ ss4. I don’t feel guided by my supervisor.

Job demands

▪ jd1. I’m pressured to work full hours.
▪ jd2. I have unreasonable deadlines.
▪ jd3. I am forced to neglect some tasks because they involve too much work.
▪ jd4. Different work groups require tasks that are difficult to perform together.
▪ jd5. I can’t take adequate breaks.
▪ jd6. My workload is excessive.

Job autonomy

▪ ja1. I can decide when to take a break.
▪ ja2. I can plan or schedule the work.
▪ ja3. I have a certain margin of options to decide what to do.
▪ ja4. I have a certain amount of options to decide how to proceed with my work.
▪ ja5. I have full autonomy to choose the methods to use to complete the work.

Colleague’s support

▪ cs1. I receive the help and support I need from my colleagues.
▪ cs2. I receive the respect I deserve from my colleagues.
▪ cs3. My colleagues are willing to listen to work related problems.
▪ cs4. It is difficult for me to receive concrete help from my colleagues in a difficult time.
▪ cs5. I can hardly say that my colleagues are really competent

Role ambiguity.

▪ ra1. I am clear about what is expected of me at work.
▪ ra2. I am clear about the objectives and goals of my department/office/team.
▪ ra3. I know what I have to do to do my job properly.
▪ ra4. I am clear about my responsibilities and my duties.
▪ ra5. I understand how my work depends on the general objectives of the organization.

Innovation

▪ In1. Creating new ideas for difficult issues (idea generation).
▪ In2. Searching out new working methods, techniques, or instruments (idea generation).
▪ In3. Generating original solutions for problems (idea generation).
▪ In4. Mobilizing support for innovative ideas (idea promotion).
▪ In5. Acquiring approval for innovative ideas (idea promotion).
▪ In6. Making important organizational members enthusiastic for innovative ideas (idea promotion).
▪ In7. Transforming innovative ideas into useful applications (idea realization).
▪ In8. Introducing innovative ideas into the work environment in a systematic way (idea realization).
▪ In9. Evaluating the utility of innovative ideas (idea realization).
